# Identifying Barriers and Facilitators to Pain Management With Buprenorphine for Patients With Kidney Failure: A Thematic Analysis of Interviews With Key Partners

**DOI:** 10.1016/j.xkme.2025.101130

**Published:** 2025-09-30

**Authors:** Cramer J. Kallem, Megan E. Hamm, Flor Cameron, Kerri L. Cavanaugh, Nwamaka D. Eneanya, Hailey W. Bulls, Caroline Wilkie, Karlyn A. Edwards, Donna M. Olejniczak, Jane M. Liebschutz, Manisha Jhamb

**Affiliations:** 1 Department of Medicine, University of Pittsburgh, Pittsburgh, PA; 2 Division of Nephrology, Department of Medicine, Vanderbilt University Medical Center, Nashville, TN; 3 Department of Medicine, Emory University, Atlanta, GA; 4 Punta Gorda, FL

**Keywords:** Pain, hemodialysis, stigma, buprenorphine, qualitative

## Abstract

**Rationale & Objective:**

Treating pain with analgesic medications in patients with kidney failure receiving dialysis is often complicated owing to altered pharmacokinetics, affecting efficacy and safety of medications. Buprenorphine, a partial opioid agonist without these limitations, has potential value in pain treatment in kidney failure. The aim of this qualitative study was to identify barriers and facilitators to overcoming stigma for implementation of buprenorphine as a pain treatment in patients treated with dialysis.

**Study Design:**

Thematic analysis of interview data.

**Setting & Participants:**

We used snowball sampling to enroll physicians with expertise in pain management and kidney failure, and other key partners.

**Exposures:**

Participants completed semistructured interviews. Topics covered included facilitators and barriers to pain management and buprenorphine prescription for patients with kidney failure.

**Outcomes:**

Qualitative themes.

**Analytical Approach:**

Interviews were transcribed and coded using the MAXQDA software (VERBI Software). We performed a thematic analysis to determine the most salient themes.

**Results:**

Of the 26 participants, 17 were physicians with expertise in nephrology, addiction psychiatry, palliative care, internal medicine, and physical medicine and rehabilitation. The other 9 participants included representatives from payors, retail pharmacy, and dialysis organizations. We identified the following 5 themes: (1) lack of knowledge about buprenorphine, (2) pervasiveness of pain- and buprenorphine-related stigma, (3) perception of pain management as beyond nephrologists’ scope of practice, (4) sociostructural barriers to pain management, and (5) suggestions to overcome the barriers.

**Limitations:**

Our sample had limited racial and ethnic diversity and may not represent the perspectives of key partners working within different health systems or geographic locations.

**Conclusions:**

Significant barriers to effective pain management and access to buprenorphine exist for patients with kidney failure at multiple levels (ie, patient, provider, organization, and systems levels). Therefore, multilevel interventions that include components that target patient stigma, clinician education, and increase collaboration among key partners are needed.

## Plain-Language Summary

Buprenorphine has potential value and safety in pain treatment in kidney failure. The aim of this qualitative study was to identify barriers and facilitators to overcoming stigma for implementation of buprenorphine as a pain treatment in patients treated with dialysis. We interviewed 26 participants with expertise in pain management and kidney failure, which revealed the following themes: (1) lack of knowledge about buprenorphine, (2) pervasiveness of pain- and buprenorphine-related stigma, (3) perception of pain management as beyond nephrologists’ scope of practice, (4) sociostructural barriers to pain management, and (5) suggestions to overcome the barriers. Because the identified barriers exist at the patient, provider, organization, and systems levels, multilevel interventions that include components that target patient stigma, clinician education, and increase collaboration among key partners are needed.

Patients with kidney failure have a high symptom burden.^1^, ^2^, ^3^ Pain is among the most common and debilitating symptoms experienced by these patients^3^, ^4^, ^5^ and has been associated with lower health-related quality of life, increased psychosocial distress, presence of other concurrent symptoms (eg, depression, fatigue, and poor sleep), and poorer survival.^6^, ^7^, ^8^ Pain also has negative implications for health costs as it has been associated with poorer adherence to dialysis, increased emergency department visits, inpatient hospitalizations, and a greater length of stay.^8^^,^^9^

Despite its negative consequences, pain is frequently underrecognized and undertreated in patients with kidney failure.^10^, ^11^, ^12^ A number of barriers at the patient, provider, and health-system level likely contribute to these suboptimal outcomes. For example, the pain experiences of patients with kidney failure are highly heterogenous, which makes treatment selection challenging.^11^^,^^13^ Furthermore, there are relatively few analgesic medications that have demonstrated efficacy in patients with kidney failure, and pharmacotherapy is often complicated by the pharmacokinetic effects of dialysis, increased risk of toxicity, and polypharmacy.^13^^,^^14^ Patients also face barriers to accessing pain management services (eg, time constraints owing to lengthy dialysis appointments, reduced contact with primary care providers after initiating dialysis, and shortage of pain management providers).^13^^,^^15^, ^16^, ^17^

Owing to the limited availability of effective treatment options for chronic pain, patients with kidney failure have high opioid use, despite having an elevated risk of opioid-related complications.^13^^,^^18^, ^19^, ^20^ Buprenorphine, a synthetic opioid that acts as a partial μ-opioid receptor agonist, is an attractive treatment option for pain in kidney failure, owing to its lower risk profile and because its pharmacokinetics are not affected by hemodialysis.^21^^,^^22^ In addition, unlike other opioids, buprenorphine is primarily excreted through the liver, so it can be administered in normal doses in this population without risking accumulation of the drug or its metabolites.^23^ Although patients with kidney failure are rarely prescribed buprenorphine, prescription rates have steadily increased from 0.1% in 2011 to 0.5% in 2020, whereas prescription rates of other opioids have decreased.^24^

Barriers to the use of buprenorphine have been most frequently studied in the setting of opioid use disorder.^13^^,^^25^, ^26^, ^27^ Buprenorphine is currently approved by the Food and Drug Administration for pain prescribed by any clinician with a Drug Enforcement Agency license, although there is some variability in specific state regulations and its current principal use is treating opioid use disorders.

Besides cost, insurance regulations, and unfamiliarity with the medication, “stigma” may decrease its use in appropriate pain conditions.^28^, ^29^, ^30^ Chronic pain- and opioid-related stigma is believed to limit access to pharmacotherapy for pain management through a number of pathways^31^ including increasing physician hesitancy in prescribing opioids,^32^, ^33^, ^34^ pharmacist vigilance in dispensing opioids,^28^^,^^35^ and patient fear of judgment when discussing treatment options for their pain.^30^^,^^32^ Stigma may have a particularly strong influence on access to buprenorphine for pain management owing to its association with opioid use disorder,^36^ which has been documented in methadone^37^ and naloxone.^38^

The aim of this qualitative study was to conduct semistructured interviews with physicians and other key partners to identify barriers and facilitators to overcoming stigma for implementation of buprenorphine as a pain treatment in patients with kidney failure receiving dialysis.

## Methods

### Setting and Participants

This was an ancillary study of the Hemodialysis Pain Reduction Effort Consortium trial, a randomized control trial to test the effectiveness of a behavior-based treatment strategy on pain and opioid use in 640 patients receiving hemodialysis with chronic pain.^39^ Participants were recruited for the current study between June 2021 and December 2021 using a snowball sampling approach. Hemodialysis Pain Reduction Effort Consortium members were asked to identify potential participants to gather a broad range of relevant perspectives including physicians from diverse professional backgrounds as well as representatives from insurance companies, retail pharmacy, and large dialysis organizations. Because sample size in qualitative studies is driven by the concept of “saturation,” defined as the point at which collecting additional data does not yield additional insights, and observational studies have shown that saturation can occur in as few as 6 interviews but is likely to occur between 12 and 16 interviews,^40^ our initial sampling goal was 30 participants (20 clinicians and 10 nonclinicians including pharmacists, payers, and those representing large dialysis organizations). This study was approved by the institutional review board at the University of Pittsburgh and all participants provided informed consent.

### Data Collection and Analysis

Data collection and analysis were conducted by experienced qualitative analysts at the Center for Biostatistics and Qualitative Methodology’s Qualitative Core, under the direction of a medical anthropologist qualitative methodologist (MEH). Participants were asked to provide demographic information after completion of their interviews. Trained qualitative researchers (FC, MEH, and JW) conducted one-on-one in-depth, semistructured interviews (intended to last for 30-60 minutes) by phone or Zoom videoconferencing; FC was the primary interviewer, with JW assuming interviewing responsibilities if FC was unavailable. Topics covered were facilitators and barriers to pain management and buprenorphine prescription for patients with kidney failure, as well as participants’ experiences with policies related to the treatment of pain in this population, which were contextualized based on their professional role (Items S1-S4). Interviews were transcribed verbatim, and a codebook was developed using an inductive approach by the primary coder (FC). Data collection concluded when the interviewers determined that saturation had been reached, with the note that there were too few interviews from nonclinicians to have saturation by subtype of participant (ie, the distinct perspectives of pharmacists vs large dialysis organization representatives), whereas acknowledging that the nonclinician interviews as a whole told a cohesive story. Interviews were then coded by 2 experienced coders (FC and JW), and all disagreements were discussed and resolved between the 2 coders. All coding was completed using MAXQDA 2022 (VERBI Software). The primary coder (FC) then conducted a thematic analysis, following the steps laid out by Braun and Clark.^41^ These completed analyses were then reviewed with the other members of the study team who are content experts, who helped contextualize the themes within the existing kidney failure, stigma, and pain literatures.

## Results

### Sample Characteristics

**Figure 1:**
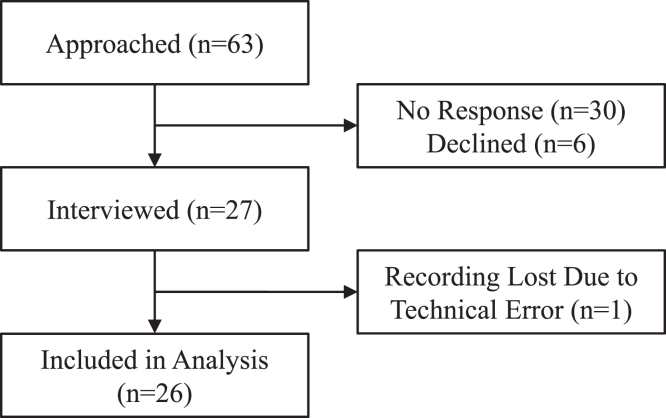
Participant flow diagram.

**Figure 2:**
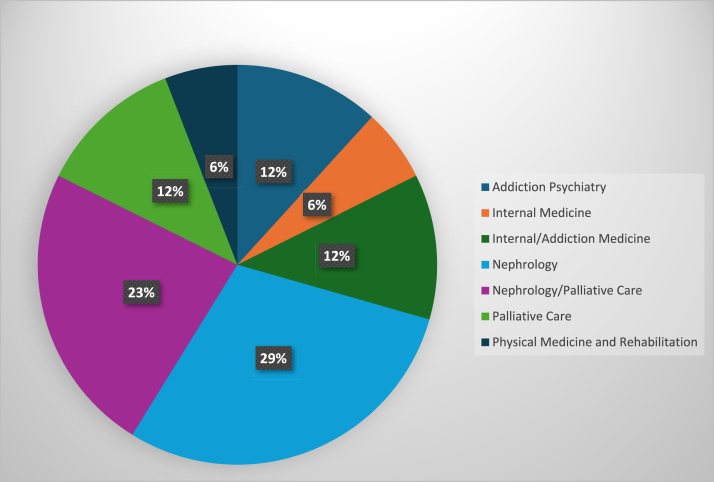
Specialties of physician participants.

**Figure 3:**
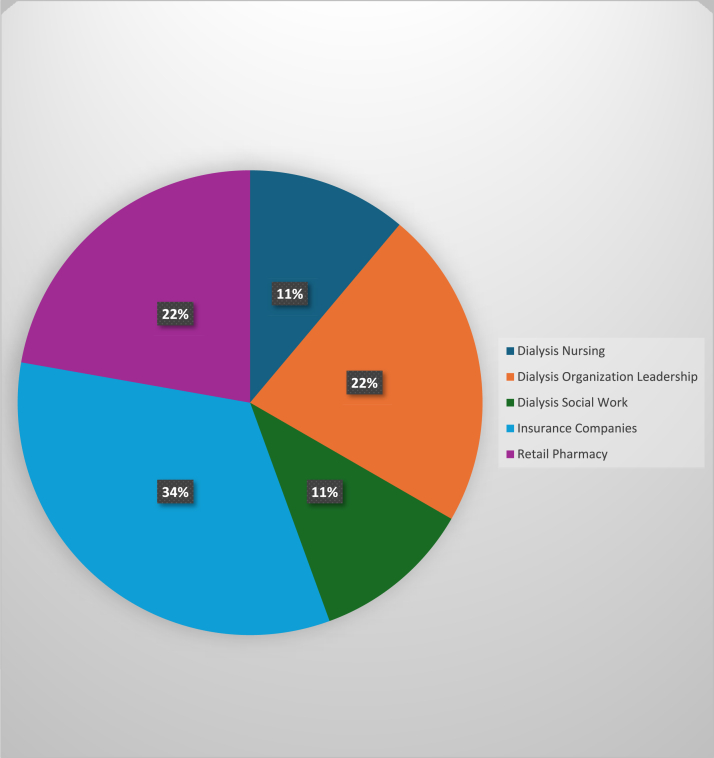
Specialties of nonphysician participants.

A total of 26 participant interviews (physicians n = 17; key partners n = 9) were analyzed (Fig 1). We successfully recruited participants from diverse professional backgrounds, including physicians with various specialties (ie, nephrology, addiction psychiatry, palliative care, internal medicine, and physical medicine and rehabilitation; Fig 2) as well as representatives from retail pharmacy, insurance companies, and dialysis organizations (Fig 3). For the 18 participants who provided demographic information, the mean age was 44 years (standard deviation = 11.17) and the majority were women (n = 12, 66.7%) and of non-Hispanic White race (n = 15, 83.3%) (Table 1).

**Table 1:** Sample Characteristics

Characteristic	Total (n = 26)
Age^a^, y (mean ± SD)	44.4 ± 11.7
Sex^a^	
Women, n (%)	12 (66.7)
Men, n (%)	6 (33.3)
Race^a^	
Asian, n (%)	3 (16.7)
White (non-Hispanic), n (%)	15 (83.3)
Physicians (n = 17)	
Addiction psychiatry, n (%)	2 (7.7)
Internal medicine, n (%)	1 (3.8)
Internal/addiction medicine, n (%)	2 (7.7)
Nephrology, n (%)	5 (19.2)
Nephrology/palliative care, n (%)	4 (15.4)
Palliative care, n (%)	2 (7.7)
Physical medicine and rehabilitation, n (%)	1 (3.8)
Other representatives (n = 9)	
Dialysis nursing, n (%)	1 (3.8)
Dialysis organization leadership, n (%)	2 (7.7)
Dialysis social work, n (%)	1 (3.8)
Insurance companies, n (%)	3 (11.5)
Retail pharmacy, n (%)	2 (7.7)

Abbreviation: SD, standard deviation.

a Descriptive statistics are based on the 18 participants who provided demographic information. Demographic information was not collected from 8 participants owing to time constraints during their interviews.

### Thematic Analysis

The following 5 themes were identified: (1) lack of knowledge around buprenorphine, (2) pervasiveness of pain- and buprenorphine-related stigma, (3) perception of pain management as beyond nephrologists’ scope of practice, (4) sociostructural barriers to pain management, and (5) suggestions to overcome the barriers.

#### Theme 1: Lack of Knowledge Around Buprenorphine

A key barrier discussed around pain management in general and more specifically around buprenorphine prescription was the lack of knowledge among clinicians about buprenorphine prescribing. Many participants commented on the paucity of clinical guidelines on the use of buprenorphine for pain management. One physician participant explained:

“(There are) misperceptions about whether or not you can use (methadone- and buprenorphine-based products …) during dialysis. The good news … is that neither is dialyzable, so there’s no need (to add) medication back after dialysis. So, it is actually pretty straightforward.” – P7 (Addiction Psychiatry)

However, the physician participant did acknowledge there are factors that can make buprenorphine prescribing more complex (eg, comorbid medical conditions and polypharmacy). Although prescribers never needed a special waiver for prescribing buprenorphine for pain or opioid use disorder,^42^ it is worth noting that some participants were not aware of these regulations. Of note, as of January 2023, waivers were no longer needed for any buprenorphine prescribing. As this nephrologist stated:

“Do I have a waiver? … No, I didn’t know you even needed a waiver, so there you go (laughs) …. Probably even more reason I shouldn’t prescribe it.” – P11 (Nephrology)

Clinicians also perceived lack of knowledge among patients and reported that most patients were skeptical about how effective buprenorphine will be for pain control, perhaps because most patients only know about its use for treating opioid use disorder (Table 2).

**Table 2:** Additional Quotes Illustrating Themes

Theme	Quotes
1. Lack of knowledge around buprenorphine	“There’s a lot of guidelines around buprenorphine for opioid use disorder. I mean, there’s whole trainings about it. (… However,) buprenorphine for pain alone without co-occurring opioid use disorder, there’s not as much clinical practice guidelines.” – P8 (Internal Medicine/Addiction Medicine)
	“I know nothing about buprenorphine, I have no idea how to get education about it, and, … one is most comfortable prescribing things that they grew up with and they learned about during their training and in their practice, and when a new drug comes along, especially when it’s something like a narcotic … where there’s high risks involved. I think always difficult to plunge into an experiment with something new, … so I haven’t explored that medication at all.” – P11 (Nephrology)
	“I think some patients are not aware that it is helpful for pain and they are skeptical initially, … but often they’re pleasantly surprised when (it is effective).” – P1 (Addiction Psychiatry)
2. Pervasiveness of pain- and buprenorphine-related stigma	“There are definitely providers who have particular thoughts about both buprenorphine and methadone. I will say that, often, when they see patients improve with it, those attitudes can change. … And also, trying to reframe the way that they’re thinking about patients can be helpful too.” – P7 (Addiction Psychiatry)
	“… Often, (health care professionals) are thinking about buprenorphine or methadone as substitution therapy, and what the patient really needs to do is to stop using (opioids). Helping them see it as a therapeutic for a chronic medical problem rather than just an enabling mechanism is often the first thing.” – P7 (Addiction Psychiatry)
	“There are people that look at people that have chronic pain and they just think that they’re just whiners or they’re just drug seeking. Instead of really looking deeper at the issue to see they really have pain.” – Org 3 (Dialysis Social Work)
3. Perception of pain management as beyond nephrologists’ scope of practice	“I don’t think it’s overall been a priority for nephrology care. … If a patient has complained of pain, (most nephrologists) tell the patient that they should see their primary care physician to have them prescribe medication if necessary.” – Org 2 (Dialysis Nursing)
	“When you’re seeing a patient in the dialysis unit, … there is limited privacy, so] if a person’s having a problem with pain management, especially in the nature of addiction, that’s something that is often a private issue and not something that you’d want to have the potential for information spreading inappropriately.” – P5 (Nephrology)
	“If you did end up with a patient who was abusing a medication and you had to break the (controlled pain medication) contract with them, you still have to provide them with dialysis. Some of the dialysis units I go to, I’m the main provider in my group. So, … if a patient is feeling uncomfortable with the situation, (… they) can switch dialysis units, but it’s sort of a lot for someone, especially if that unit is just down the road for them.” – P5 (Nephrology)
	“I find that, if we manage the pain medications through someone who’s not at the dialysis unit, we can still provide our expertise, but it allows that sort of safe buffer for the patient to have good oversight and, you know, a relationship with the pain management person, but it doesn’t necessarily interfere with the dialysis management.” – P5 (Nephrology)
4. Sociostructural barriers to pain management	“We have quite a lot of prior authorization on (buprenorphine-based pain products). And also, a level limit, like 60 for 30 days for the patch, it’s 4 for 28 days, so, it is specific to the dosage form.” – Insurer 2 (Managed Care Organization)
	“I treat a lot of uninsured patients. They don’t always have access to good pain management.” – P1 (Addiction Psychiatry)
	“I had patient on dialysis with a significant social determinants of health burden—I think they were unstably housed—who had been inconsistently attending dialysis appointments which led to their [hospital admission].” – P7 (Addiction Psychiatry)
	“(Patients are) needing to be their own advocate, their own case manager, when dealing with chronic medical disease that can already affect your executive function and ability to do those things. So I think we’re asking a lot of somebody to try to navigate a disjointed system.” – P7 (Addiction Psychiatry)
	“One of the challenges in treating (patients with kidney failure) with buprenorphine, (… is we cannot perform) routine urine drug screening, so we were having to get blood draws. Blood toxicology is a little more invasive and a little less patient-centered now that we have point-of-care urine and saliva toxicology in the clinic.” – P1 (Addiction Psychiatry)
5. Suggestions to overcome the barriers	“I think what is most helpful for me as an addiction specialist would be the removal of the restrictions around prescribing it. It’s a little bit unusual to think that anybody who has prescribing power can write as much oxycodone as they want, but, not everybody can write for buprenorphine. That leads to a lot of weird processes and systems of care where you have to find the clinician in your system who’s able to write suboxone so that you can continue that medication for a patient who needs it. Sometimes it leads to systems deciding that they’re not going to offer that.” – P7 (Addiction Psychiatry)
	“I think there are knowledge gaps around which pain medications, both opioid and non-opioid, are safe and what is their hierarchy of safety … you want to have an algorithm that works for most people and points in that algorithm that direct them to involve a specialist. That can often be something that can shore up any educational gaps.” – P7 (Addiction Psychiatry)
	“I also think that having patient information that is not only focused on opioid use disorder would be very helpful to be able to give patients some information about the medication and why it can be useful for pain and not just OUD.” – P8 (Internal Medicine/Addiction Medicine)
	“We could cover (pain-specific buprenorphine products), but they’re non-preferred so they require a prior authorization. Just because there’s other pain medications that are more cost-effective and potentially more effective. (… For a drug to be listed as preferred,) we would want to see clinical evidence that it works … there does need to be some peer-reviewed research.” – Insurer 3 (State Medicaid Program)
	“…If we’re bringing our own biases or stigma, that, gets attached to these patients sometimes, both on the dialysis side, but also on the pain and opioid use disorder side, (communicating) that those can be effectively managed, usually that is going to improve things. ‘Cause bias and stigma can definitely produce blind spots.” P7 (Addiction Psychiatry)

#### Theme 2: Pervasiveness of Pain- and Buprenorphine-Related Stigma

Participants acknowledged that there is considerable stigma around pain management and particularly around prescription pain medication. One clinician said:

“I think because of current stigmas, some providers, make it a rule to not prescribe (opioids) at all, which I don't feel is fair, particularly for the patients.” – P12 (Nephrology/Palliative Care)

Some of the clinicians who prescribed buprenorphine shared experiences in which their colleagues expressed stigmatizing attitudes about buprenorphine:

“I’ve certainly had (colleagues) who don’t know a lot about (buprenorphine) make assumptions about it. They would say things like, wow, are you really prescribing that? Or, you know, this patient’s just an addict.” – P7 (Addiction Psychiatry)

One discussed how the association of buprenorphine with opioid use disorder may contribute to the stigma around prescribing the drug to manage pain:

You know, we had an interesting phenomenon—when we started prescribing buprenorphine, our nursing staff in our clinic had such a hard time understanding the difference between the pain-specific products and suboxone. … We’ve never had our nurses be so uncomfortable about the clinicians prescribing a drug as they have been about buprenorphine. … They’ve had such an amplified response. – P14 (Palliative Care)

#### Theme 3: Perception of Pain Management as Beyond Nephrologists’ Scope of Practice

Although pain is recognized as an important and salient issue for patients with kidney failure, many of the health care professionals who work with these patients do not consider pain management to be part of their scope of practice or priority. As one participant put it:

“Since we are focused on their kidney disease and providing them life-saving treatment, I don’t really feel like we do a good job of managing their pain. Since that’s not really our primary purpose in treating them.” – Org 3 (Dialysis Organization Social Work)

Other participants expressed concerns that dialysis clinics are not an appropriate setting for pain management services owing to the lack of privacy and felt that patients should be referred to a specialist. A nephrologist participant noted that the long-term nature of patient-nephrologist relationships may in fact complicate pain management if the nephrologist wants to break the pain contract owing to suspected medication abuse, but still needs to continue to provide dialysis care (Table 2). More often, nephrologist participants reported some degree of comfort managing pain, but only in specific contexts. For example, only pain related to the dialysis procedure:

I tend to shy away from managing pain outside of something that is obviously acute and obviously related to (my scope). So, if somebody has pain with cannulation or they’ve got a lot of swelling or discomfort, then I tend to address that right then and there, but I very much shy away from treating chronic pain in my CKD and especially my dialysis patients, just by principle. – P2 (Nephrology)

Of note, we did find one exception to this theme, which was as follows: physician participants who specialized in both nephology and palliative care felt it was their responsibility to manage pain for their patients with kidney failure, given the high symptom burden associated with chronic pain. As one physician participant expressed:

“I feel like there’s a cohort of (chronic pain) patients in nephrology that often get abandoned, and those are the patients that I will definitely take on (but …) it can’t just be one person handling these patients.” – P15 (Nephrology/Palliative Care)

#### Theme 4: Sociostructural Barriers to Pain Management

The most frequently cited structural barrier to pain management was the high appointment burden, which all patients receiving hemodialysis face.

“When the patient is having to go to dialysis a few days a week, having an additional mental health or pain medicine appointment, can just complicate their life.” – P1 (Addiction Psychiatry)

Several participants discussed how social determinants of health-related factors such as financial resource strain, housing instability, and lack of transportation can further limit patients’ access to pain management services outside the dialysis clinic. One participant also described how structural racism may play a role in suboptimal pain care:

“I think that the other barrier is that our patients tend to have encountered a lot of structural racism and a lot of difficulty with access to healthcare and been not treated well by the system at all. There’s a lot of skepticism as far as their degree of pain and what their experience is.” – P16 (Palliative Care)

Participants also noted factors specific to kidney failure and policy structural issues, which limit patients’ access to buprenorphine. For example, these patients are not able to provide urine samples for periodic urine drug monitoring, which is a standard component of care when prescribing opioids for the management of chronic pain (Table 2).^43^ Several participants commented on the ways in which insurance companies’ policies can be a barrier to patients accessing pain medication. As one clinician put it,

“I find that it’s more of a fight than it should be to try to get people good pain control. We have a patient who we were (prescribing) opioids for, and their insurance company is saying they won’t pay for more than 5 days of the opioid … so, the patients have to go to the pharmacy every 5 days to get it for the first 5 weeks.” – P16 (Palliative Care)

#### Theme 5: Suggestions to Overcome the Barriers

Participants proposed several strategies to improve pain management outcomes in patients with kidney failure. Some discussed strategies they used to reduce stigma. For example, one physician participant highlighted the importance of recognizing and managing one’s individual biases and considering how one’s communication can either reduce or perpetuate sigma (Table 2).

With regard to increasing access to buprenorphine, participants provided the following 2 recommendations: (1) removing the unique restrictions around prescribing it (this has since been accomplished with the elimination of the DATA-Waiver Program^42^) and (2) developing specific prescribing algorithms for managing pain in patients with kidney failure (Table 2).

Several clinicians discussed the benefits of a multidisciplinary, multimodal approach to pain management (Table 2). However, care coordination can be challenging; as this physician participant highlighted, patients often experience:

“Disconnected care, where they are working with multiple providers who are often not in direct communication or within the same electronic health care record.” To which they proposed the following solution: “Intensive case management where we are helping the patients with their organization and getting to appointments and transportation is really what is needed.” – P7 (Addiction Psychiatry)

Another participant expressed optimism that nephrologists could help overcome this barrier and be effective in pain management:

“Nephrologists are the ones who see the patients most often, more than their primary care physicians or anybody else. Because they … have access to the patients on a frequent basis, I think nephrologists potentially could be the ones, and feel comfortable to take a more active role in pain management.” – Org 4 (Dialysis Organization Leadership)

This dialysis organization representative suggested that building partnerships between dialysis organizations and pain management organizations could also improve patient outcomes.

“We recognize that we need to manage quality of life issues to help patients have the best outcomes. So, we’ve had the opportunity through some of the value-based care arrangements to be more formal with partnering with palliative care.” – Org 1 (Dialysis Organization Leadership)

## Discussion

In a sample of key partners from diverse professional backgrounds, significant barriers to buprenorphine prescription and pain management for patients with kidney failure were identified. We found that most dialysis clinicians did not consider managing chronic pain to be their responsibility, with the only exceptions being primary care providers, palliative care physicians, and those with dual certifications in nephrology and palliative care. This aligns with previous research showing nephrologists tend to view pain management as the responsibility of nonnephrology providers.^44^

Whereas the majority of patients with chronic pain without kidney failure receive pain management services from their primary care provider,^45^ most hemodialysis patients would prefer their nephrologist and dialysis care team provide comprehensive care.^15^^,^^46^ This is particularly relevant for pain management because access is limited to specialty clinics owing to the severe shortages in both palliative care^16^ and pain medicine^17^ specialists. Coordinating referrals and appointments with any health care specialist is challenging owing to the high appointment burden throughout the typical week, and sociostructural barriers this population faces (eg, area-level deprivation and low individual financial, social, and transportation resources).^47^, ^48^, ^49^

National nephrology organizations, large dialysis organizations, and health policy leaders will need to convene to determine “who” should be managing chronic pain in hemodialysis patients and “how” to overcome access and sociostructural barriers. Possible strategies could include integrating specialty services (eg, pain management, behavioral medicine, palliative care, addiction psychiatry) within dialysis clinics. Newer value-based models of care such as collaborative nephropsychology that provide integrated evidence-based behavioral medicine services (see Jhamb et al^50^ for an example) within the dialysis clinic need to be tested to determine implementability and sustainability.^51^ Nephrology teams in dialysis clinics can play a unique role in comanaging pain because of their frequent contact with the patient. They can be trained to take shared responsibility in diagnosing, referring, care coordination, ensuring treatment adherence, and monitoring safety and treatment response. Moreover, increasing the dialysis team’s awareness of the pain undertreatment that these patients face may help increase their willingness to participate in pain comanagement. However, policy-level changes are needed to prioritize and align pain management with quality metrics and financial incentives in dialysis care delivery. It will also be important to consider how integrating pain management in dialysis care could affect the nephrologist’s workload, as they often have competing demands and priorities to address dialysis-related quality metrics, and have a high rate of burnout as a result.^52^ Leveraging existing care team model in a dialysis clinic including nurses and social workers to address pain will be critical for real-world feasibility of pain management in dialysis.

An important solution to addressing some of the challenges is incorporation of training and development of clear clinical guidelines for the management of chronic pain in patients with kidney failure. This may increase nephrologist comfort and acceptance of responsibility for managing chronic pain, as was evident from nephrologists trained dually in palliative care. Training on safety and efficacy of pain medications, alongside understanding of regulations around buprenorphine use such as the need for a Drug Enforcement Agency license, should be an integral part of early nephrology fellowship training. Nephrology fellowships should also teach strategies to overcome issues related to privacy within the dialysis unit and manage potential conflicts that can arise with pain management contracts (ie, for opioid prescriptions) in the context of long-term nephrologist-patient relationships.

The need for pain management guidelines specific to kidney failure is necessary considering the heterogenous pain experiences, altered pharmacokinetics affecting medication safety and efficacy, and effect of coexisting untreated symptoms such as depression and anxiety on pain. Unfortunately, relatively few randomized controlled trials of pain management interventions for patients with kidney failure have been conducted,^13^^,^^53^^,^^54^ and more studies are needed to provide a scientific basis for best practice guidelines. Given the pharmacokinetic properties of buprenorphine, research testing its efficacy in patients with kidney failure should be prioritized.^21^^,^^22^

In addition to developing comprehensive clinical guidelines, it will be important to consider potential barriers to implementation and sustainability. Our findings indicate that pain- and opioid-related stigma may present a profound barrier to adoption of best practices. The existence of pain-related stigma, which is often compounded by systemic racism in health care is well documented.^55^, ^56^, ^57^ Although participants acknowledged the existence of stigma, they did not endorse personally holding stigmatizing attitudes. It is possible that some clinicians and key partners may not recognize the implicit bias they hold or the ways they may be unintentionally perpetuating stigma,^58^^,^^59^ or that individuals willing to participate in this study were atypical in their attitudes. Thus, increased training on recognizing and addressing stigma is needed for all health care providers (see Heijnders and Van Der Meij^60^ for a review of stigma reduction strategies). There is a growing body of evidence to support the use of multilevel interventions to address stigma in health care.^61^ Because the barriers identified in our study exist at the patient, provider, and health-system levels, multilevel interventions are needed to make meaningful progress in improving the pain management outcomes for patients with kidney failure.

Study limitations include a snowball sampling approach, which resulted in a sample with limited racial or ethnic diversity and may not represent the perspectives of key partners working within different health systems or geographic locations. Furthermore, study sample size constrained achievement of saturation of themes “within” each physician specialty and key partner type. It is also important to note that participants were not specifically asked to comment on the feasibility of integrating pain management services into dialysis clinics. Considering efforts to prioritize pain management in dialysis settings could potentially burden the nephrology workforce, a more targeted assessment of feasibility would be needed before implementing this model.

Despite these limitations, this study has several strengths. To our knowledge, this is the first qualitative study to examine barriers to buprenorphine prescription for pain management in patients with kidney failure from the perspective of key partners with a wide range of professional backgrounds. We recruited a group of diverse professions including physicians with expertise in nephrology, palliative care, addiction medicine, psychiatry, and primary care as well as representatives from insurance companies, retail pharmacy, and dialysis organizations. This allowed us to analyze a broad range of perspectives and identify consequential barriers to buprenorphine prescription for pain management in patients with kidney failure. Our findings also revealed opportunities to overcome these barriers, which can be used to inform multilevel intervention development.

Future studies should test the feasibility and effectiveness of multilevel interventions to improve pain management and reduce stigma in patients with kidney failure. Interventions should use a collaborative care approach and include components that target pain-related stigma, clinician education, interdisciplinary collaboration, and strengthening relationships among key partners. These interventions should be informed by implementation science and consider the sociostructural barriers this population faces to maximize the potential for adoptability and sustainability.

## Supplementary Materials

Supplementary File (PDF) Items S1-S4.
