# Supplementary material for: Identifying Barriers and Facilitators to Pain Management With Buprenorphine for Patients With Kidney Failure: A Thematic Analysis of Interviews With Key Partners
Source: Kidney Med. 2025 Sep 30;7(12):101130. doi: 10.1016/j.xkme.2025.101130 (PMC12664035; doi:10.1016/j.xkme.2025.101130)
Supplement: Supplementary File (PDF) — Items S1-S4. [file mmc1.pdf]

Item S1. Interview Script for Physician Participants

1. I would like to start by asking about your experience with providing pain management for patients on dialysis/with chronic kidney disease. Tell me about your experience doing this.

- Probes if needed:
  - Are you the only physician managing their pain?
  - Are you the primary manager of your patients' pain? If not, who is?
  - If others are involved in pain management, who is that? How do you communicate with that team?
  - What challenges do you face?
  - Is there anything that improves pain management for these patients, or makes it easier?

2. Now, I would like to ask about any experience you may have prescribing opioids and buprenorphine/suboxone.

- Let's start with opioids. Do you prescribe opioids for any patients in your practice?
  - If Yes: Tell me about the contexts in which you prescribe them.
    - Do you ever prescribe them for patients on dialysis/with chronic kidney disease? Why or why not?
      - Tell me about the most recent patient on dialysis/with chronic kidney disease for whom you prescribed an opioid. What factors contributed to your prescribing them? How effectively did they manage your patients' pain?
  - If No: Tell me a little bit about why not.
    - Probes only if necessary: Are opioids not relevant/necessary? Are they difficult to prescribe?
- OK, now let's talk about buprenorphine/suboxone. Do you prescribe buprenorphine/suboxone for any patients in your practice?
  - If Yes: Tell me about the contexts in which you prescribe it.
    - Do you ever prescribe it for patients on dialysis/with chronic kidney disease? Why or why not?
      - Tell me about the most recent patient with kidney disease for whom you prescribed buprenorphine. What factors contributed to your prescribing it?
        - If it was prescribed for pain: How effectively did it manage your patient's pain?
      - Have you experienced any challenges in prescribing buprenorphine (or opioids) for patients on dialysis/with chronic kidney disease, who also have pain?
        - How do your patients react when you suggest buprenorphine for kidney pain?
        - What is most difficult for you? For your patients? Can you tell me more about that? Can you give me an example?

        - Have you ever felt judged, pressured, or shamed for prescribing, or not prescribing buprenorphine (or opioids)? Tell me more about that.
- If No: Tell me a little bit about why not.
    - Are you waivered to prescribe buprenorphine?
        - If not waivered: Tell me a bit about the reasons you aren't waivered.
        - If waivered: If you were treating a patient on dialysis/with chronic kidney disease who you felt could benefit from buprenorphine, would you prescribe it yourself or refer them to someone else? Tell me about the factors that would contribute to your decision.
        - For all: How do you think a patient on dialysis/with chronic kidney disease might react if you were to suggest using buprenorphine for their pain?
- For all: Does the association of buprenorphine with treatment for opioid use disorder affect your decision-making around prescribing it for chronic pain? If so, how?
- For all: Does the association of buprenorphine with treatment for opioid use disorder affect how your patients think about taking it for chronic pain? If so, how?

Now we're going to switch gears and talk a little bit about the opioid epidemic and some social determinants of health.

3. Tell me about how the opioid epidemic has affected your clinical practice.
    - How has it affected the community your practice serves?

4. Do you think the opioid epidemic has influenced care for patients on dialysis/who have pain from chronic kidney disease in any way? If yes, how so?

5. There is some research to suggest that buprenorphine could be useful for pain in patients on dialysis because it is metabolized by the liver, is not dialyzed and has lower risk for sedation than other opioids such as morphine. What opportunities do you see to improve buprenorphine prescribing for patients on dialysis?
    - Tell me more about that.
    - What would be most helpful for you? For your patients?

6. Is there anything else that you think might improve the pain management of patients with chronic kidney disease?

7. Lastly, I wanted to ask about kidney care and pain management a little bit more generally. Are you aware of any racial or socioeconomic inequities in kidney care and pain management?
    - If Yes: Tell me about that. What are you aware of?

- If No: There is research that suggests that patients from marginalized racial groups and lower socioeconomic backgrounds experience more barriers to care than other patients. What is your reaction to this? Does it ring true?
- For all: What are your thoughts about whether or not these factors might affect prescription of buprenorphine for patients on dialysis?
- For all: Can you think of any ways to improve these inequities?

8. Is there anything I didn't ask about that you think we should know?

Thank you so much for your time.

Item S2. Interview Script for Retail Pharmacists

Today, we're mostly going to talk about buprenorphine prescriptions. So, to start: When you see a prescription for buprenorphine come up to be filled, what comes to mind?

- Probe if necessary:
  - Are there any interactions you check for?
  - Are there any expectations you have about the encounter with the patient?

Now, imagine that you see a prescription for buprenorphine come up to be filled for a patient that you know or suspect has end-stage renal disease. What would come to mind? Would you have any concerns?

- Probe if necessary: Has this ever happened? If so, tell me about that.
  - Probes if necessary:
    - Why was it prescribed? (For pain? For opioid use disorder?)
    - How often does it happen?
    - Do the patients have any particular questions or concerns?

Now, I'd like to ask about your general experiences in filling buprenorphine prescriptions.

- What do you think of buprenorphine as a medication? What benefits does it have? What concerns do you have with it, if any?
- What, if any, issues with patients do you encounter when filling buprenorphine prescriptions?
- What, if any, issues with providers do you encounter when filling buprenorphine prescription ?
- What, if any, issues do your pharmacy techs have when filling buprenorphine prescription (if you know)?
- What, if any, issues with insurance do you encounter when filling buprenorphine prescription ?

Lastly: there is some research to suggest that buprenorphine could be useful for pain in patients on dialysis because it is metabolized by the liver, is not dialyzed and has lower risk for sedation than other opioids such as morphine. Are you familiar with this?

- If prescribing buprenorphine for patients with end-stage renal disease became more common, what impacts would it have on you? What impacts would it have on patients?
- What, if any, policies or procedures would need to change if this happened? To the best of your knowledge, how would we go about changing them?

That was my last question. Is there anything else you think we should know? Is there anything you thought I would ask about that I didn't?

Item S3. Interview Script for Insurers

Today, we're going to be talking about your plan's current policy regarding buprenorphine prescribing. So, to begin, what is your current policy about buprenorphine prescribing?

- Probe if necessary:
  - Under what circumstances is it approved?
    - What level of expertise do the people who make decisions on coverage have? What training do they undergo?
  - Do you require prior authorization?
    - If so, how are decisions about whether to approve the prescription made?
  - What tier formulation and copay is required for the prescription?

  - Does coverage vary with different plans that you offer – i.e., private vs. Medicare or Medicaid options?

Now, I'd like to provide you with a little bit of information and then ask your thoughts about that information and how it might affect your plan's policies. There is some research to suggest that buprenorphine could be useful for pain in patients on dialysis because it is metabolized by the liver, is not dialyzed and has lower risk for sedation than other opioids such as morphine. Were you familiar with this? Does your plan currently approve the use of buprenorphine for pain? Why or why not?

- If not approved: Given the potential benefits, what would it take to change the policy to approve buprenorphine for pain in patients on dialysis?
- Would the plan consider allowing automatic authorization for patients with end-stage renal disease? What information would you need to approve this?
- What tier formulation and copay would your plan consider to encourage the use of buprenorphine in this patient population for pain?
- Currently, there are two formulations of buprenorphine that are approved for pain (Belbuca, which is an oral medication, and Butrans, which is a patch) but the higher dose forms, like Subutex or Suboxone, are approved for opioid use disorder treatment only – what would it take to change to approve higher dose forms for pain?

That was my last question. Is there anything else you think we should know? Is there anything you thought I would ask about that I didn't?

Item S4. Dialysis Organization Representatives

Today, we're going to be talking about chronic pain management in your organization. I would like to start off by acknowledging that we know that you are doing a lot of excellent work for a group of patients with very complex medical needs, that chronic pain management is only one of their needs, and that it isn't the primary purpose of the care you give. So, I'm going to be asking about how your organization handles and prioritizes chronic pain management, and what, if any, changes you might like to see in that arena, but I want to remind you that there are no right or wrong answers here – we are trying to get a sense of the issue as you experience it. Do you have any questions for me before we get started?

So, let's start with a very general question: What is your understanding of the chronic pain management issues faced by your patient population?

How does chronic pain management for patients on dialysis fit into the priorities of your organization?

What do you see as your organization's role in the chronic pain management of your patients?

- Do providers participate in chronic pain management? How so?
    - If you identified that one of your patients had chronic pain were inappropriately managed, what would you do? Are there any structures in place that would help you to address this?
- What do you think is the role of nephrologists in chronic pain management?
- How do reimbursement pressures affect your thinking about pain management?
- What do you think works well about how your organization manages patient pain, or is involved in managing patient pain?
- What, if anything, do you think could be improved about how your organization manages patient pain, or is involved in managing patient pain?
- If someone were interested in improving pain care within the organization, how do you think that could be accomplished? What would need to happen? Who would need to be on-board for it to happen?

Now, I'd like to provide you with a little bit of information and then ask your thoughts about that information and how it might affect your organization's policies. There is some research to suggest that buprenorphine could be useful for pain in patients on dialysis because it is metabolized by the liver, is not dialyzed and has lower risk for sedation than other opioids such as morphine. Were you familiar with this?

- If we wanted your organization to identify patients who might benefit from the use of buprenorphine for chronic pain management, how would we go about making that happen?
    - Would you be open to it?
    - What would need to change for this to happen?
    - What supports would you need?
- Are there other, related comorbidities or symptoms that your organization monitors or manages? If so, what are they? (If needed: What about depression?)

- If the organization monitors/manages other conditions/symptoms: What processes and policies do you have in place to monitor/manage that?
    - Could similar processes and policies be used to monitor and manage pain?
    - What would be easy about doing it that way?
    - What would be difficult? How could those processes / policies be improved for pain management?

That was my last question. Is there anything else you think we should know? Is there anything you thought I would ask about that I didn't?
